# Partially-functional exhausted CD8+ T cells can contribute to short-term viral suppression: a computational prediction for children with perinatal HIV

**DOI:** 10.1101/2025.09.18.677001

**Published:** 2025-09-20

**Authors:** Alexis Hoerter, Alexa Petrucciani, Fatma Marshed, Mussa Mwamzuka, Aabid Ahmed, Alka Khaitan, Elsje Pienaar

**Affiliations:** 1 Weldon School of Biomedical Engineering, Purdue University, West Lafayette, IN, USA;; 2 Bomu Hospital, Mombasa, Kenya; 3 Ryan White Center for Pediatric Infectious Diseases and Global Health, Indiana University School of Medicine, Indianapolis, IN, USA; 4 Regenstrief Center for Healthcare Engineering, Purdue University, West Lafayette, IN, USA.

## Abstract

We and others have reported evidence of T cell exhaustion in children with perinatal HIV with increased expression of inhibitory receptors PD-1, CD160, and TIM-3, but there is limited data on the virologic functional consequences of this immune exhaustion. We address this by using an immune database from Kenyan children with perinatal HIV and unexposed controls. We computationally integrate T cell profiles of differentiation, activation and exhaustion in an agent-based model (ABM) to predict how T cell exhaustion impacts viral control following HIV exposure in vitro. Our ABM includes macrophages, CD4 and CD8 T cells, cytokines, and HIV. Model mechanisms include viral dynamics, macrophage activation, T cell activation and proliferation, cytotoxic T cell killing, and cytokine/HIV diffusion and degradation. Participants are grouped by HIV plasma viremia and by age, less than 5 years or 5–18 years. Our findings indicate that cells from virally active participants, who have the highest levels of exhaustion, have lower predicted viral concentrations and infected cells compared to other participant groups during new infection. However, this coincides with higher cell death, suggesting that short-term viral control is associated with excessive inflammation, which could be detrimental long-term. Cells from virally suppressed participants older than 5 years can maintain lower viral concentrations while limiting cell death, reflecting a more sustainable short-term immune response. In virally suppressed children younger than 5 years, immune response patterns strongly resemble the age-matched healthy control group, suggesting early viral suppression may preserve antiviral immune responses. Our model predicts unique patterns of cell death for each participant group, with CD8 T cell death being dominant in virally active groups and CD4 T cell and macrophage death being dominant in healthy and virally suppressed groups. Finally, exhausted CD8 T cells are predicted to contribute significantly to CD8 T cell killing, proliferation, and activation in the virally active group, indicating partially functional CD8 T cells can still contribute to short-term viral control. Our analysis functionally integrates participant-specific immunophenotypic data to allow quantification of the extent, mechanisms, and impact of immune dysfunction in perinatal HIV and could inform pediatric HIV remission and cure strategies.

## Author Summary

Cytotoxic CD8+ T cells are vital for our ability to kill infected cells. However, chronic stimulation of CD8+ T cells can result in T cell exhaustion, leading to cellular dysfunction and reduced ability to fight infections. Here we ask: What impact does CD8+ T cell exhaustion have on the ability to control HIV replication over the short term?

We answer this using a computational model, anchored in experimental data from children with perinatal HIV. We use participant-specific data on dozens of immune markers that characterize the state of their immune system. We carefully map these markers to known immune cell functions and use our model to predict how immune cells with these participant-specific markers would respond to HIV infection.

Our results suggest that earlier viral suppression can lead to better immune function in children with perinatal HIV. Our findings also suggest that exhausted CD8+ T cells could still contribute to fighting HIV infection, but only if there are enough of them to compensate for their reduced functionality.

Our work uses computational models to put valuable participant-specific data into context, allowing us to predict short-term infection outcomes and better understand immune function in children with perinatal HIV.

## 1. Introduction

Despite effective antiretroviral therapy (ART) of both mothers and children, 160,000 children are infected with HIV each year(1). As in adults, ART can effectively control, but not eliminate, HIV in children. Long-term infection in children is also associated with chronic inflammation leading to T cell exhaustion and weakened immune responses to HIV. However, the extent, mechanisms and impact of this immune dysfunction in children with perinatal HIV (CPHIV) remains unclear. Immune exhaustion, and CD8 T cell dysfunction in particular, has been identified in CPHIV through elevated expression of immune inhibitory receptors (IRs) such as PD-1, TIM-3, 2B4 and CD160. We and others have reported elevated PD-1 levels on CD8 T cells in CPHIV, but there is limited data on other IRs and their functional consequences(2–5). In a South African child with sustained virologic remission, the one distinct immune feature was high PD-1 levels, warranting further investigation into PD-1 as a biomarker of clinical outcomes or treatment interruption in children(6).

Collection and analysis of pediatric samples to answer these questions are challenging. Pediatric study participants are more difficult to recruit than adult participants(7). If samples can be obtained from children, the smaller amounts of blood that can be safely and feasibly collected from children limit the types of analyses that are possible. For example, functional assays such proliferation assays can only be performed on total PBMCs since the small sample volumes are insufficient to sort immune subsets. These bulk analyses therefore also pose an analysis challenge in quantifying the relative contribution of individual immune cell subtypes.

Here we address these cohort building and data analysis challenges by pairing clinical parameters, immune phenotypic data and *in vitro* functional assays with computational systems biology approaches. We leverage an established database of clinical and immunologic parameters from a cohort of CPHIV and a control group of children unexposed to HIV (CHU) (3,5,8–13). We couple these participant-specific data with a mechanistic computational model that emulates *in vitro* HIV infection assays. Our established agent-based models (ABMs) (14–16) provide virtual versions of *in vitro* HIV infection assays, incorporating viral, innate and adaptive immune dynamics. These computational models, parameterized with participant-specific data, complement their experimental counterparts by allowing quantitative characterization of complex host-pathogen interactions with high spatial and temporal resolution.

Here, we use our computational models to predict the impact of exhausted CD4 and CD8 T cells (expressing PD-1, TIM-3, and/or CD160) on short-term viral control and immune responses to new HIV exposure. Our simulations provide testable predictions and generate new hypotheses about the functional impact of T cell exhaustion on HIV-specific immune responses. Our findings serve to integrate complex clinical and immune datasets, predict host responses to viral blips and inform future HIV functional cure approaches.

## 2. Materials and Methods

### 2.1 Cohort and Immune Phenotypic Data:

We utilize an established database from the Pediatric Immune Activation (PIA) study as described previously(3,5,8–13). Briefly, a total of 242 children between the ages of 2 months to 20 years were enrolled from Bomu Hospital, an HIV-centered nonprofit hospital in Mombasa, Kenya from 2011–2012. The cohort was comprised of 156 CPHIV, including 76 children who were treatment naïve, and 84 children who had been on antiretroviral therapy for at least six months. As controls, 82 children unexposed to HIV (CHU) were enrolled.

Blood samples were collected from all children to perform HIV viral loads and CD4 cell counts and to isolate and cryopreserve PBMCs and plasma. For each participant, we performed a broad immune phenotype analysis by flow cytometry to delineate innate (monocytes, dendritic cells, natural killer cells) and adaptive immune subsets (B, Th1, Th2, Th17, Th22, regulatory T cells), T cell differentiation states (naïve, central, transitional and effector memory and EMRA), chemokine receptor expression, T cell activation (CD38, HLA-DR) and exhaustion (PD-1, CD160, TIM3, 2B4), and proliferative (Ki67) and cytotoxic (granzyme B, perforin) potential. Flow gating strategies are detailed in (3,5,8). The immune phenotypic data was compiled with clinical data including complete blood counts, HIV viral load, CD4 T cell counts, and antiretroviral treatment to build a master de-identified database of clinical and immune parameters.

In total, 116 participants have complete immune phenotypic profiles for simulation parameters discussed below in Section 2.2.9 and are used in simulations. CPHIV was divided into groups based on viral suppression for analysis in our computational models of HIV infection dynamics. CPHIV virally suppressed (CPHIV-VS) participants are defined as those with HIV viral load less than 200 copies/mL and CPHIV virally active (CPHIV-VA) are defined as those with viral loads equal to or above 200 copies/mL. Table 1 shows the demographic and clinical characteristics of the participants used in simulations.

### 2.2 Simulation Description:

**Fig. 1:**
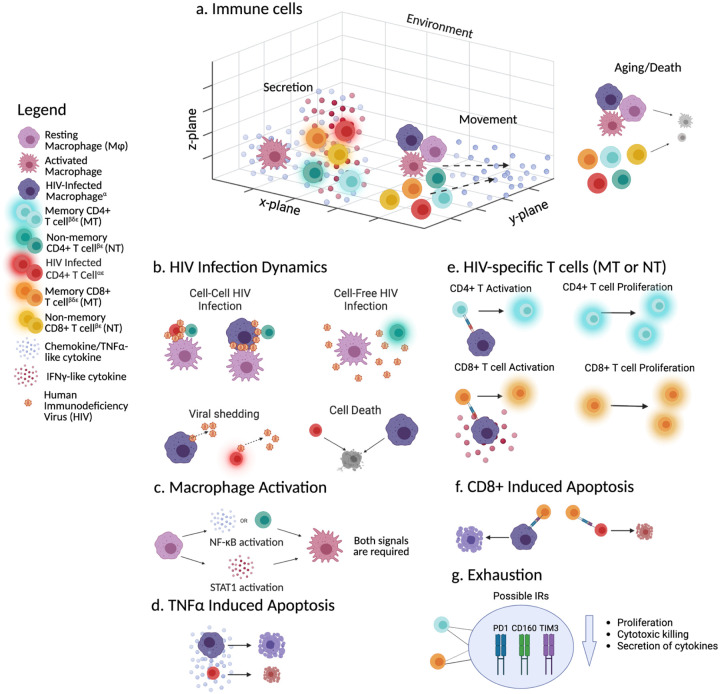
Schematic of general ABM agents and rules adapted from(14–16). CD4 and CD8 T cells are divided into two main populations: memory and non-memory. a) All immune cells exist in a 3D environment and remain at the bottom z-layer unless on top of another cell. Each compartment can hold only one immune cell. Only activated or recently differentiated CD4 T cells and CD8 T cells secrete TNFα and IFNγ (cell must be TNFα/IFNγ+) and activated macrophages secrete TNFα. All immune cells move probabilistically along the TNFα concentration gradient. All immune cells have ages (resting and activated) and die when their maximum lifespan is reached. b) HIV can infect activated and CCR5+ CD4 T cells or macrophages either via cell-free concentration (HIV >1) or through infected cell contact. Only activated HIV-infected cells shed virus. After a maximum infection time, HIV-infected cells die. c) Macrophages can become activated by activating both NF-κB and STAT1 pathways. d) Any cells can apoptose based on the local concentration of TNFα. e) HIV-specific CD4 and CD8 T cells can become activated/differentiated and can divide. CD8 T cells need the HIV-infected cell to be STAT1 activated. f) CD8 T cells can kill (apoptose) HIV-infected cells (if memory must be HIV-specific and Granzyme+ or Perforin+; if recently differentiated effector must be HIV-specific. g) Up to three IRs (PD1, CD160 or TIM3) can be found on memory CD4 (blue) and CD8 T (orange) cells that can decrease their proliferation probabilities, cytotoxic killing and secretion of cytokines (IFNγ and TNFα). The severity of the loss of function is determined by the number of IRs on the cell. α: Must be CCR5+; β: Can be HIV-specific; δ: Can have IRs; ε: Glow represents activated cell (memory) or recently differentiated effector cell (non-memory); +: Only activated cells at the start can perform any functional operations. After initialization, only HIV-specific cells can perform functions once activated. See Figure 2 for flowchart of possible markers that can be found on T cells. Created in Biorender. Created in BioRender. (2025) https://BioRender.com/t09v629

**Fig. 2:**
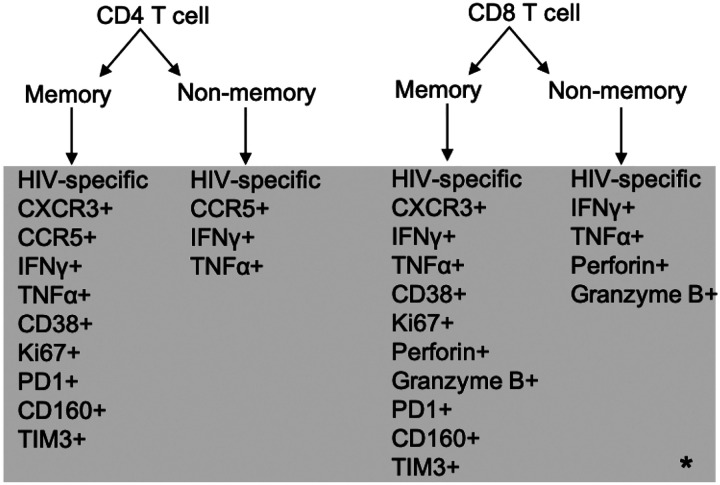
Flowchart of the various clinical markers that can be assigned to CD4 and CD8 T cells in our simulation. *: the markers in the grey box are randomly assigned in the simulation to their given population, resulting in a simulated population of cells with a heterogeneous total number of markers per cell. Meaning any given cell can have any number of the markers listed under its respective subtype. It’s important to note that if a memory non HIV-specific T cells starts activated (Ki67+ or CD38+) then it can perform cytokine secretion (if IFNγ+ and/or TNFα+), help activate macrophages and kill infected cells if a CD8 T cell (only if granzyme B+ or perforin+) otherwise it does nothing but move regardless if it is positive for other markers. Non-memory HIV-specific cells can become activated by interacting with HIV infected cells, but none start activated. These cells can also be positive for other markers, but if they are not HIV-specific they will never be activated to perform any of the functions those markers are associated with.

Our ABM uses unique, individual participant immune phenotypic data from the cohort described in Section 2.1 to predict the immune response to new HIV infection *in vitro*. For simulations, we group participant data by HIV RNA copies/mL at the time of data collection: HIV RNA copies/mL = 0 (CHU), < 200 (CPHIV-VS), and ≥ 200 (CPHIV-VA) as well as by age (0–5 years, and ≥ 5 years). We use the same model rules as described in (14–16), and briefly summarized below, but with the initial parameters described below as well as mechanism changes to reflect HIV infection. The ABM consists of macrophages, CD4 and CD8 T cells, two diffusible cytokines TNFα and IFNγ, and HIV. All cell types have their own rules that are executed simultaneously mimicking realistic cell behavior. General model mechanisms are described in Fig. 1. A detailed flowchart of possible clinical markers for T cells is given in Fig. 2. An outline of the general clinical markers mapped to simulation mechanisms is given in Table 2, and model parameters are given in Table 3. We simulate an *in vitro* HIV infection for 14 days with a timestep of 6 minutes, approximately the time it takes for a macrophage to move its own length (17–20) (20 μm) or one grid square(14). The simulation is implemented in Java using Repast Simphony (21). MATLAB and Python are used for data analysis and visualization. We use ChatGPT to create and edit graphing scripts for MATLAB, and to summarize methods text from (14–16) for the model mechanisms that are not changed in this work. Any content generated by ChatGPT was carefully reviewed by our team for accuracy.

#### 2.2.1 Environment:

The ABM environment is a 3D grid with 100 × 100× 10 compartments, representing a 2 mm × 2 mm × 0.2 mm subsection of a well-mixed *in vitro* HIV infection assay (e.g. a well in a 96 well plate). Each grid compartment is a cube with a width of 20 μm, with toroidal (x-y plane) and no flux (z plane) boundaries (Fig. 1a).

#### 2.2.2 Cytokines:

We represent IFNγ and TNFα as continuous variables and we numerically solve partial differential equations describing their diffusion and degradation using a 3D alternating-direction explicit method (14,22,23).

#### 2.2.3 Immune Cells:

All immune cells (macrophages, CD4 and CD8 T cells) move by following a TNFα chemotactic gradient, and can “fall” due to simulated gravity. Movement is probabilistic and based on cells’ current functional state, resting or activated (14–16). In brief, cells will generally move towards higher concentrations of TNFα. The concentration of TNFα in each grid in the surrounding Moore neighborhood are summed up and then each grid’s individual TNFα concentration is divided by the sum of the concentration in the Moore neighborhood to give a probability of moving to that grid square. See (14) for more details regarding movement rules. Cell aging accelerates upon activation, and TNFα-induced apoptosis is simplified from (24) and described in Equation 1 in Supplement Materials.

#### 2.2.4 Macrophages:

HIV-infected macrophages, representing all antigen-presenting cells, activate HIV-specific T cells, approximating MHC II and I antigen presentations(25). Macrophages secrete TNFα upon activation through both NF-κB, induced by TNFα or CD4 T cell contact, and STAT1 pathways induced by IFNγ (26–29).

#### 2.2.5 T cells:

CD4 and CD8 T cells in the simulation are divided into 2 subtypes: memory and non-memory. We describe the characteristics of each subtype below, along with the markers from the immune phenotypic database that are used to parameterize these subtypes in the model. Table 2 provides a mapping of clinical markers to simulation mechanisms and Fig. 2 shows a flowchart of the possible clinical markers that can be found on simulated T cells. These clinical markers were determined for certain cell populations and not for their single-cell combination, therefore the combinations of markers per cell is random. **Table 1** in Supplement Materials provides a more detailed clinical marker to simulation parameter mapping.

##### 2.2.5.1 Functions assigned to memory CD4 T cell phenotypes:

Memory CD4 T cells in the simulation are characterized by expression of immune markers for movement (CXCR3+), infection susceptibility (CCR5+), cytokine secretion (IFNγ+, TNFα+), activation (CD38+), proliferation (Ki67+), HIV specificity, and inhibitory receptors (PD1+, CD160+, and TIM3+ (Section 2.2.7)) based on the immune phenotypic data. The chemokine receptor CXCR3 has been identified to have an impact on cellular movement and CXCR3+ cells are 5–10 fold more efficient at moving (30,31). To keep things as simple as possible, all cells are initially assigned base and activated movement probabilities according to our estimated parameter ranges. If a cell is CXCR3+ then a random number between 5 and 10 is sampled and multiplied to the base and activated movement probabilities. Equation 2 in Supplement Materials demonstrates how this is calculated for a cell. This results in most CXCR3+ cells being guaranteed to move at every timestep. Proliferated cells that are CXCR3+ will sample new movement probabilities when they are created. CCR5+ cells represent cells that are susceptible to HIV infection as this is one of the main coreceptors necessary for HIV entry into cells. Only cells positive for IFNγ will secrete IFNγ if activated. Likewise, only cells positive for TNFα will secrete TNFα if activated. We use the CD38+ marker to determine the number of cells that start the simulation already activated. We use the Ki67+ marker to determine the number of cells that are already at some random point in their proliferation cycle and also activated. For the purpose of our simulation, we assume that CD38+ and Ki67+ cells represent different pathways of activation and thus do not overlap these markers. Therefore, only the memory cells that are Ki67- will be allowed to be CD38+. Cells that start with the Ki67 marker will have their current “generation” sampled between (1,maximumCD4Generations). HIV-specific CD4 T cells can become activated by randomly selecting and interacting with a macrophage near them. If the macrophage is HIV-infected, the T cell has some probability of becoming activated, reflecting MHC II antigen presentation(25). Activation is not for an indefinite time period; CD4 T cells have a chance to deactivate each timestep which is an approximation for anti-inflammatory mechanisms. Any activated CD4 T cell can replicate within a variable timeframe up to a generational limit (maximumCD4Generations) and will secrete IFNγ and TNFα at defined rates if positive for those markers. Activated CD4 T cells can also help macrophages get NF-κB activated [see [(14–16) for more details]. Memory CD4 T cells can have 0, 1, 2, or 3 IRs, which impact their other functional abilities (discussed below in 2.2.7). The frequency of these markers is determined by individual participant data, but co-expression of all these markers is randomly assigned per cell during simulation initialization due to a lack of combination staining. Resting HIV-specific and non-specific CD4 memory T cells move around the simulation without further action unless they are CCR5+, in which case they can be infected. The only action non-specific CD4 memory T cells can perform is cytokine secretion but only if activated from the beginning.

##### 2.2.5.2 Functions assigned to memory CD8 T cell phenotypes:

Memory CD8 T cells are characterized by expression of immune markers for movement (CXCR3+), cytokine secretion (IFNγ+, TNFα+), activation (CD38+), proliferation (Ki67+), cytotoxic killing (perforin+, granzyme B+), HIV specificity, and exhaustion (PD1+, CD160+, and TIM3+ (Section 2.2.7)) based on the immune phenotypic data. We assume the percentage of CXCR3+ memory CD8 cells is similar to that of CD4 memory cells, due to lack of data for the CD8 T cell population. The framework for CXCR3+, IFNγ+, TNFα+, CD38+, and Ki67+ for memory CD4 T cells (see 2.2.5.1) applies to memory CD8 T cells as well. Memory CD8 T cells that are either perforin+ or granzyme B+ act as cytotoxic T cells, and if they are HIV-specific, can probabilistically kill HIV-infected cells. HIV-specific CD8 T cells can become activated by finding an HIV-infected macrophage that is also STAT1 activated which acts as a proxy for co-stimulation(32) reflecting MHC I antigen presentation(25). Activated HIV-specific CD8 T cells can kill HIV-infected cells (CD4 T cell or macrophage) with some probability, secrete TNFα and IFNγ if positive for those markers, and proliferate in the same way as CD4 T cells. Memory CD8 T cells can have 0, 1, 2, or 3 IRs which impact their other functional abilities, if also positive for those markers, which is discussed below in 2.2.7. The frequency of these markers is determined by individual immune phenotype, but the co-expression of all these markers is randomly assigned per cell during simulation initialization due to a lack of combination staining. Resting HIV-specific and non-specific CD8 memory T cells move around the simulation without further action. The only action non-specific CD8 memory T cells can perform is cytokine secretion but only if activated from the beginning.

##### 2.2.5.3 Non-memory T cells:

T cells that are not memory subtypes are considered naïve T cells at the start of the simulation with some portion being HIV-specific. Naïve CD4 and CD8 T cells with HIV-specific markers can be primed within the simulation upon encountering HIV-infected macrophages, turning into recently differentiated effector T cells. Additional immune markers in this recently differentiated effector T cell population include cytokine secretion (IFNγ+, TNFα+) and cytotoxic killing (perforin+, granzyme B+) (CD8 only). These recently differentiated effector T cells can secrete cytokines (only if positive for IFNγ or TNFα), proliferate, activate macrophages (CD4 only) and kill HIV-infected cells (only perforin+ or granzyme+ CD8s). These cells, which cannot become exhausted in the simulation, exhibit movement probabilities akin to CXCR3- cells. Their TNFα and IFNγ secretion rates, along with proliferation and killing probabilities (CD8), are determined as for non-exhausted cells (Equation 1). All other naïve T cells, move around the simulation without further action unless they are CCR5+ (CD4), in which case they can be infected.

#### 2.2.6 HIV Specificity:

In a subset of participants, the frequency of HIV-specific T cells was identified by *ex vivo* proliferation assays as previously described(3,5). Since proliferation assays were not performed for all participants, we set the value of HIV-specific CD4 and CD8 T cells to be 2% for every participant.

#### 2.2.7 Markers of T cell Exhaustion:

The immune database includes the frequency of PD1+, CD160+, and TIM3+ CD4 and CD8 T cells, however, the explicit function of each of these IRs is unclear. Prior studies show that CD8 T cell dysfunction or exhaustion increases with co-expression of multiple IRs(5,33). We assumed that the number of IRs displayed on each cell correlates linearly with the degree of exhaustion, at least for CD8 T cells (5). It is unknown whether this is true for CD4 T cells, but we will assume for our purposes here that it is similar. Therefore, we will employ a binning approach linking IR co-expression count to exhaustion severity, affecting functional parameters like proliferation rate and cytokine secretion in CD4/CD8 T cells, and CD8 T cell killing probability (Fig. 1g). Equation 1 demonstrates how the number of IRs impacts the decrease in proliferation parameters through the use of a random multiplier. Secretion and killing parameters are determined in the same way. We assume IRs are inherited upon cell proliferation(33,34). A new multiplier for each parameter is randomly selected based on the number of IRs for the newly proliferated cell. Cells cannot become newly exhausted in our simulations as the time scale we are evaluating is too short.

Equation 1: Assuming a linear correlation with the number of IRs expressed on a cell with more severe exhaustion, we developed a multiplier strategy to determine exhausted cell parameters for proliferation, secretion (TNFα/IFNγ), and CD8 T cell killing. Here we show an example for how these multipliers are utilized for the number of IRs for T cell proliferation (CD4 and CD8). CD4/CD8 T cell IFNγ secretion rate, CD4/CD8 T cell TNFα secretion rate, and CD8 T cell killing probability are calculated the same way. Cells with 3 IRs will have the most severe loss to their functional parameters. For example, if the probability of proliferation is sampled at 75% for the population of CD8 T cells then a single CD8 with 3 IRs will have a proliferation probability of no more than 75%*10% = 7.5%. For 2 IRs, it will be between 0% and 15%, and for 1 IR, 0% to 22.5%. We allow for heterogeneity by making each possible multiplier of IRs not just one multiplier but a range. This way not all cells with the same number of IRs are identical in their function. The ranges we selected also mean that a cell with a single IR could have a multiplier smaller than a cell with 3 IRs.

$$P_{\text{proliferation}}=\left\{\begin{array}{l} P_{\text{base}\_\text{proliferation}}\times R\sim U(0,0.1);3\;\text{IRs}\\ P_{\text{base}\_\text{proliferation}}\times R\sim U(0,0.2);2\;\text{IRs}\\ P_{\text{base}\_\text{proliferation}}\times R\sim U(0,0.3);1\;\text{IRs}\\ P_{\text{base}\_\text{proliferation}}\times R\sim U(0.9,1);0\;\text{IRs} \end{array}\right.$$

#### 2.2.8 HIV Infection:

In our model, HIV is a diffusing entity where free virus spreads and degrades. Only activated CCR5+ CD4 T cells and macrophages can be infected probabilistically, either by free virus or cell-to-cell transmission (35,36). Immune cells randomly try free virus or cell-to-cell infection first, and if unsuccessful, the second method will be tried. For free virus infection, the virus concentration at the grid location of the cell needs to be at least 1 and a random number check against the probability of free virus infection needs to be successful. Cell-to-cell infection requires an infected neighbor and a successful probability of cell-to-cell infection check against a random number.

Infected cells enter an eclipse phase before they can productively shed virus; however, only activated cells can shed virus, at different rates for macrophages and CD4 T cells. If an activated HIV-infected CD4 T cell proliferates, then the progeny will also be HIV-infected(37). HIV-infected cells have an average lifespan of 2.2 days(38). We initialize the simulation with one large amount of virus (4.94e+04 ± 3.05e+04 [mean±std]) in the center of the grid. This ratio of virus to target cells (macrophages and CD4s) results in a large multiplicity of infection (MOI) (22 ± 22.6). While we recognize this relatively high MOI is not a common practice in *ex vivo* experimental HIV infections, we assume it is sufficient for our purposes here because experiments usually stimulate cells (with IL-2) before HIV infection due to the challenging nature of artificially creating HIV-infected cells(35,36). In the process of identifying parameters, we discovered that the low number of initially activated cells in our simulations was a major hurdle in having an infection establish itself at lower MOIs. However, to maintain the participant-specific nature of our simulations we do not inflate the number of activated cells and assumed a high MOI of virus to cells instead.

#### 2.2.9 Initial Conditions and First Estimate Parameter Ranges:

**Table 1** in Supplement Materials shows a detailed list of the participant-specific markers incorporated into simulations. We exclude any participant from the full PIA study that was missing data for any of the immune markers in **Table 1** in Supplement Materials. Given the remaining participant-specific data (116 participants), we determine the absolute number of PBMCs per individual by subtracting the absolute neutrophil count from the white blood cell count (**WBC_x10.9.L** – **Absolute_Neu_x10.9.L**) to get the absolute number of PBMCs. Next, we divide the absolute number of lymphocytes (**Absolute_Lymph_x10.9.L**) and the absolute number of monocytes **(Absolute_Mono_x10.9.L**) by the absolute number of PBMCs we calculated to get the percentage of lymphocytes and monocytes per individual.

To assess the impact of IRs, we map model parameters to corresponding participant-specific markers where available (33 participant specific parameters) and sample other parameters within biologically feasible ranges (41 parameters). To find biologically feasible values for non-participant-specific parameters, we first used Latin Hypercube sampling (LHS)(39) on the parameters in Table 3 marked with an asterisk. We generated 580 samples with 3 replicates each to give 5 unique parameter sets per participant in triplicate (1740 total runs). We ensure each participant has a baseline number of PBMCs that is high enough to cover all specific cell categories, except for triple exhausted CD4 or CD8 T cells. Using this LHS sampling approach with the strategy discussed in 2.2.7 and Equation 1 for parameters affected by exhaustion, we selected biologically feasible parameter combinations by identifying simulations that reliably produced a robust range of HIV-infected macrophages and CD4 T cells and selected 20 representative parameter sets from these. We simulated these 20 parameter sets for each participant in triplicate for a total of 6960 runs (116 participants × 20 parameter sets × 3 replicates). Table 3 shows the initial parameter ranges used to determine the 20 parameter sets in the analysis. The final 20 parameter sets are provided in Supplement Materials with the 41 parameters that are varied between sets and constant parameters. The full 116 participant parameters are provided in the Supplement as well. Analysis was completed on the average of the 3 replicates reducing the total simulation size to 2320. The size of each group is as follows: CHU_<5_=240, CHU_≥5_=400, CPHIV-VS_<5_=260, CPHIV-VS_≥5_=380, CPHIVVA_<5_=480, and CPHIV-VA_≥5_=560. Two-tailed ttests were completed between each group for statistical analysis.

## 3. Results

### HIV status and age independently affect immune responses to new HIV exposure

**Fig. 3:**
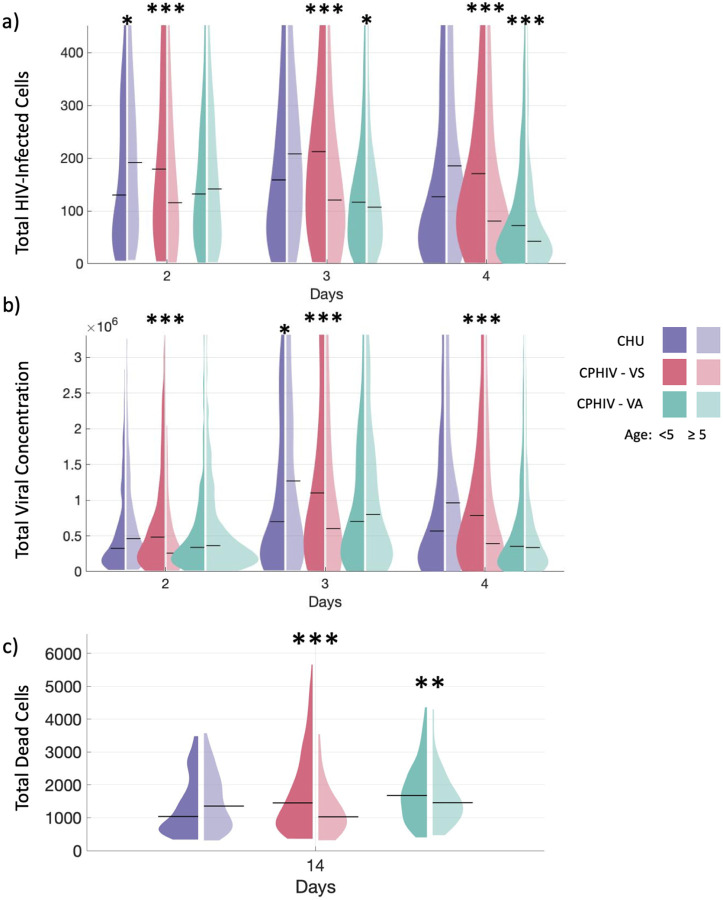
HIV status and age independently affect immune responses to new HIV exposure. a) Total HIV-infected cells for Days 2–4 post infection, b) total viral concentration for Days 2–4 post infection, c) total cell death at Day 14 post-infection. Left violin plots: participant age < 5, right: participant age ≥ 5. CHU: 0, CPHIV-VS: < 200 and CPHIV-VA: ≥ 200 HIV RNA copies/mL. From left to right n=240, 400, 260, 380, 480, 560. Violin plots show the distribution for each participant group, with black line indicating median. Y-axis for (a) and (b) are set to the 3^rd^ quartile for visibility, the full y range is shown in Fig S1 in Supplement Materials. ***p <= 1e-4, **p<=1e-3, *p<=0.01. Significance is determined with a two tailed t test between age groups for each viral participant group with α= 0.01 shown on the graph.

To predict the overall impact of participant-specific immune profiles on infection progression, we simulate our virtual *in vitro* infections and quantify viral infection dynamics (Fig. 3a,b) and cellular death (Fig. 3c). We show Day 2–4 post-infection to evaluate HIV infection dynamics as this is the peak of HIV viral concentrations and infected cells. HIV-infected cell numbers and viral concentration rapidly decline after Day 4 and are nearly zero by Day 10.

Cells from participants under 5 years of age (CHU_<5_) consistently have less virally infected cells, lower viral concentrations and less total cell death compared to cells from participants over 5 years of age (CHU_≥5_), however, majority are not significantly different (purple Fig. 3a–c). Conversely, CPHIV-VS shows statistically significant differences between age groups (pink Fig. 3a–c) (p < 1e-4, Table 4) in all of these metrics but with older participants (CPHIV-VS_≥5_) controlling new infection better. Cells from participants under 5 years of age (CPHIV-VS_<5_) are consistently predicted to have higher numbers of HIV-infected cells and viral concentration. Furthermore, this group exhibits a significantly higher number of cell death compared to CPHIV-VS_≥5_ together suggesting less effective immune responses to new viral exposure in younger compared to older CPHIV-VS participants. Patterns between age groups within CPHIV-VA vary across outputs and timepoints (green Fig. 3a–c) but the differences are small. These findings indicate that age-specific impacts vary between participant groups depending on HIV status and viral suppression. CPHIV-VS participants appear to have the largest differences in viral concentration and cell death between age groups.

For all participants under 5 years of age (darker left side of plots), the infection dynamics over a three-day period demonstrate a consistent trend (left plots Fig. 3a–c). CPHIV-VS_<5_ consistently has higher HIV-infected cells and virus concentration compared to CHU_<5_ and CPHIV-VA_<5_. CPHIV-VA_<5_ has lower viral load and HIV-infected cells (p < 1e-4, Table 4), and more cell death compared to CHU_<5_ (p < 1e-4, Table 4). This pattern suggests that for cells from participants under 5 years of age, CPHIV-VA_<5_ control viral loads to lower levels (Fig. 3a,b), but that this is associated with increased cell death (Fig. 3c). These patterns could indicate excessive inflammation which would impact the ability of CPHIV_<5_ to control infection longer term. In contrast, CPHIV-VS_<5_ simulations predict higher viral concentrations and infected cell numbers (Fig. 3a,b), but are also associated with higher cell death similar to CPHIV-VA_<5_ Fig. 3c. This suggests that there are key differences in the cell death mechanisms between virally suppressed and virally active CPHIV that result in different impacts on the viral concentration.

For participants over 5 years of age (lighter right side of plots), predictions show a more varied response Fig. 3a–c. Most groups are statistically significantly different from the other groups in number of infected cells, viral concentration and cell death (right violin plots in Fig. 3 a–c) (p < 1e-4, Table 4). For most time points, viral concentration and infected cells are lowest in CPHIV-VS_≥5_ and CPHIV-VA_≥5_ (Fig. 3a,b), and cell death is lowest in CPHIV-VS_≥5_ (Fig. 3c). These more varied patterns suggest more complex underlying mechanisms between the participant groups. However, our results indicate that cells from CPHIV-VS_≥5_ can maintain lower viral loads while also limiting cell death, which could reflect an effective short-term immune response.

### HIV status drives differential cell death patterns

**Fig. 4:**
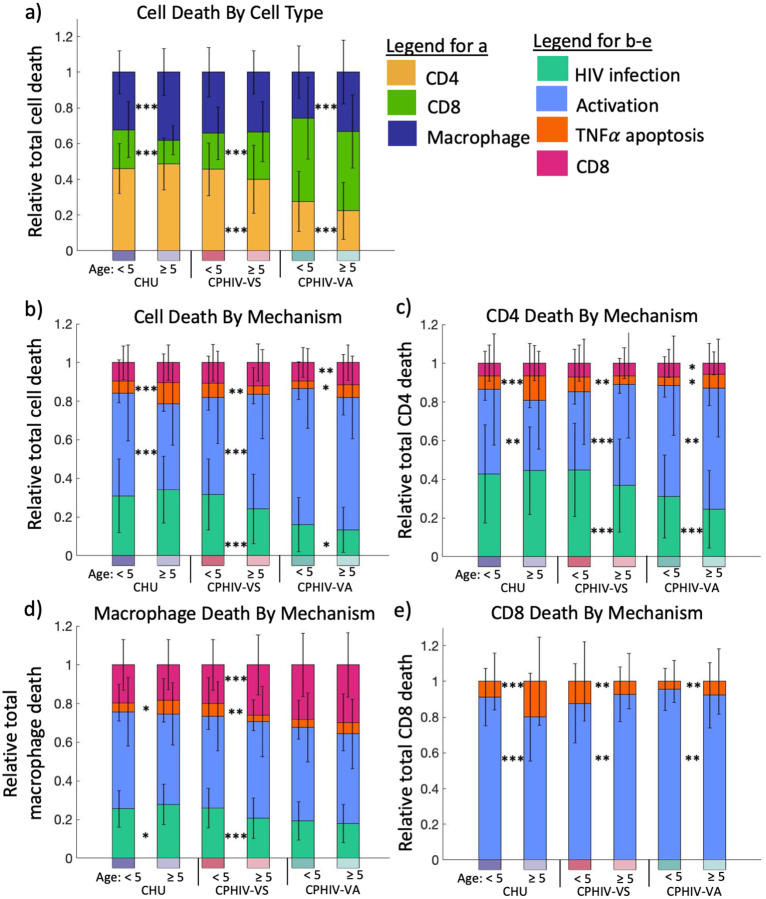
HIV status drives differential cell death patterns. a) Relative total death by cell type (bottom to top: CD4 T cell, macrophage, CD8 T cell). b) Relative total cell death by death mechanism (bottom to top: HIV infection, excessive activation, TNFα induced apoptosis and CD8 T cell killing). c) Relative CD4 T cell total death by mechanism (bottom to top: HIV infection, excessive activation, TNFα induced apoptosis and CD8 T cell killing). d) Relative macrophage cell death by mechanism (bottom to top: HIV infection, excessive activation, TNFα induced apoptosis and CD8 T cell killing). e) CD8 T cell death by mechanism (bottom to top: excessive activation, TNFα induced apoptosis). Timepoint = day 14. Bars display the mean for each group with error bars showing the standard deviation. CHU: 0, CPHIV-VS: < 200 and CPHIV-VA: ≥ 200 HIV RNA copies/mL. From left to right n=240, 400, 260, 380, 480, 560. ***p <= 1e-4, **p<=1e-3, *p<=0.01. Significance is determined with a two-tailed t test between age groups for each viral participant group with α= 0.01 shown on the graph and for age-matched groups given in Table 2 in Supplement Materials.

While CPHIV-VS_<5_ and CPHIV-VA have increased predicted cell death compared to the other groups (Fig. 3c), these overall metrics could mask underlying differences in the types of cell death. A quantification of the cell types that contribute most to this overall cell death number reveals that CHU and CPHIV-VS have similar distributions: CD4 T cells make up the largest proportion of cell deaths (~50%), followed by macrophages, then CD8 T cells (Fig. 4a). In contrast, in CPHIV-VA simulations, CD8 cells make up the largest proportion of cell deaths (Fig. 4a), almost 50% of all cell death compared to ~25% or less for CHU and CPHIV-VS (p < 1-e4, **Table 2** in Supplement Materials). CHU_≥5_ and CPHIV-VA_≥5_ show reduced death of CD8 and CD4 T cells, respectively, compared to CHU_<5_ and CPHIV-VA_<5_ (Fig. 4a). This reduction in adaptive immune cell death is associated with an increase in macrophage death in both CHU_≥5_ and CPHIV-VA_≥5_ cohorts compared to their corresponding younger age groups. Thus, our simulations indicate that HIV status drives different cell death patterns by cell type, and that older age groups (CHU_≥5_ and CPHIV-VA_≥5_) have increased cell death in the innate immune cell populations.

The proportions of cell death by different mechanisms (HIV infection, excessive activation, TNF-induced apoptosis or CD8 cytotoxic killing) provide further insight into the impacts of HIV status and age (Fig. 4b). Cell death from excessive activation (represented by Ki67+ and CD38+) is the dominant cell death mechanism in all participants groups. CHU and CPHIV-VS have significantly more deaths from HIV infection compared to CPHIV-VA (p < 1-e4, **Table 2** in Supplement Materials). CPHIV-VS shows the largest age-dependent differences between cell death mechanisms, with older participants having a larger proportion of cell deaths from excessive activation, and smaller proportions due to HIV infection and TNFα-induced apoptosis compared to the younger participants. This contrasts with CHU, where the older participants have a smaller proportion of cell deaths due to excessive activation compared to younger participants. Thus, while CPHIV-VS_<5_ have similar cell death numbers to CPHIV-VA (Fig. 3c), CPHIV-VS_≥5_ participants have similar cell death mechanism patterns to CPHIV-VA (Fig. 4b). These similarities in cell death mechanisms between CPHIV-VS_≥5_ and CPHIV-VA could help explain why these groups also have similar predicted viral load and infected cells (Fig. 3a,b), despite differences in cell death numbers (Fig. 3c).

An evaluation of cell death mechanisms for each immune cell type (Fig. 4c) further reinforces these observations and identifies how death mechanisms change for different cell types in each of the participant groups. Excessive activation remains the dominant cell death mechanism within each cell type, except for HIV-induced CD4 T cell death in CHU_≥5_ and CPHIV-VS_<5_. The overall pattern of high proportions of cell death from HIV infection in CHU and CPHIV-VS_<5_ (Fig. 4b) is present in both CD4 T cells and macrophages compared to CPHIV-VA (Fig. 4c,d) (p < 1-e4, **Table 2** in Supplement Materials). The age-dependent differences between CPHIV-VS are present in CD4 T cells, CD8 T cells and macrophages, with the most notable differences in excessive activation deaths in CD4 and CD8 T cells (again closely resembling patterns in CPHIV-VA).

Taken together, these results reveal unique patterns for each participant group both in which cell types are dying as well as how they are dying in response to short-term exposure to new HIV infection. These patterns are a result of the complex underlying composition of the immune cell population.

### Partially functional exhausted CD8 T cells contribute to anti-viral immune response

**Fig. 5:**
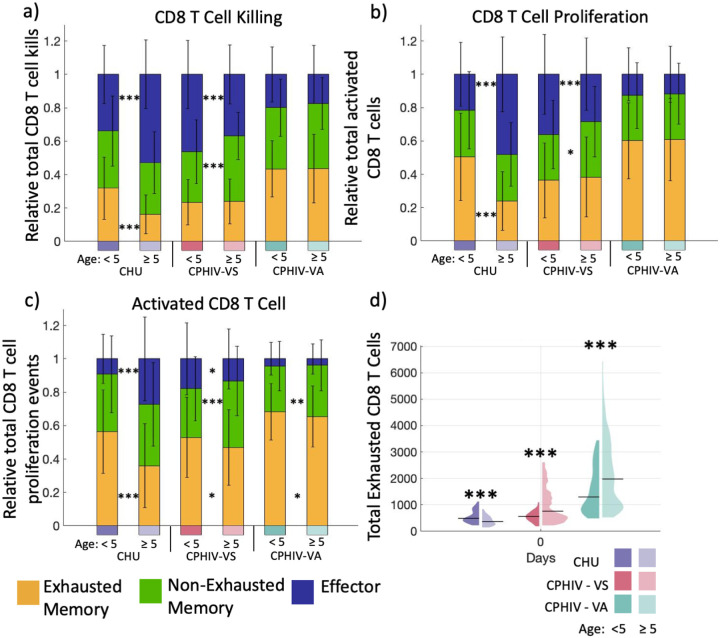
Partially functional exhausted CD8 T cells contribute to anti-viral immune response. a) Relative contributions by exhausted memory, non-exhausted memory, and recently differentiated effector CD8 T cells towards total CD8 T cell killing HIV-infected cells Day 14. b) Relative contributions by exhausted memory, non-exhausted memory, and recently differentiated effector CD8 T cells towards total CD8 T cell proliferation events by Day 14. c) Relative contributions by exhausted memory, non-exhausted memory, and recently differentiated effector CD8 T cells of total activated CD8 cells at Day 4. d) Total exhausted memory CD8 T cells at Day 0. Bars display the mean for each group with error bars showing the standard deviation. CHU: 0, CPHIV-VS: < 200 and CPHIV-VA: ≥ 200 HIV RNA copies/mL. From left to right n=240, 400, 260, 380, 480, 560. ***p <= 1e-4, **p<=1e-3, *p<=0.01. Significance is determined with a two-tailed t test between age groups for each viral participant group with α= 0.01 shown on the graph and for age-matched groups given in Table 3 in Supplement Materials. Violin plots show the distribution for each group and category with black line indicating median.

To better understand the role of exhausted CD8 T cells in the complex populations of immune cells from each of the participants, we quantify the contribution of exhausted CD8 T cells to key CD8 T cell functions during infection. Exhaustion affects CD8 T cell functions such as cytotoxic killing, proliferation, activation and TNFα and IFNγ secretion. Thus, we determined what proportion of each of these functions are performed by recently differentiated effector T cells, non-exhausted memory, and exhausted memory CD8 T cells in our simulations (Fig. 5a–c).

For CHU_≥5_ and CPHIV-VS_<5_, recently differentiated effector HIV-specific CD8 T cells kill the most HIV-infected cells (Fig. 5a). However, recently differentiated effector CD8 T cells contribute the least to CD8 T cell activation (Fig. 5b) for all age and viral concentration groups. In contrast, CPHIV-VA simulations show that the majority of CD8 T cell killing, proliferation and activation is from exhausted memory CD8 T cells (Fig. 5a–c). This indicates that while exhausted memory CD8 T cells are dominant in these CD8 T cell functions in CPHIV-VA simulations, the functions are more distributed among CD8 T cell subtypes in CHU and CPHIV-VS. However, it is important to note that the average number of total CD8 T cell proliferation events is less than 160 across all groups and the median is less than 25 across all groups with no significant differences between ages (**Fig. S2** in Supplement Materials). CHU_≥5_ consistently show that recently differentiated effector CD8 T cells contribute more (and exhausted cells contribute less) to killing (Fig. 5a), proliferation (Fig. 5b) and activation (Fig. 5c) as compared to the younger age group.

We believe that this counterintuitive prediction that the functionally impaired exhausted memory CD8 T cells are responsible for such a large proportion of CD8 T cell functions is related to the absolute numbers of exhausted cells. Indeed, CPHIV-VA simulations have higher numbers of exhausted CD8 T cells compared to CHU and CPHIV-VS (Fig. 5d) (p < 1-e4, **Table 3** in Supplement Materials), in agreement with the larger contribution of exhausted CD8 T cells to all CD8 T cell functions in CPHIV-VA (Fig. 5a–c) (p < 1e4, **Table 3** in Supplement Materials). The opposite can be seen in CHU_≥5_ participants which have the fewest numbers of exhausted memory CD8 T cells compared to their younger participants at the start of the simulation (Fig. 5d), which coincides with the larger contribution of recently differentiated effector cells towards killing, proliferation and activation (Fig. 5a–c).

Taken together, our results indicate that despite significant T cell exhaustion in CPHIV-VA, these partially functional exhausted cells can continue to contribute to the anti-viral immune response during short-term infection.

## 4. Discussion

Our study, using computational models of short-term *in vitro* exposure of participant-specific cells to HIV, reveals counterintuitive findings regarding the potential contribution of exhausted T cells to anti-HIV immune responses in CHU and CPHIV. Cells from CPHIV-VA, despite showing the highest levels of exhaustion, exhibit lower model-predicted viral concentrations and fewer infected cells upon new HIV exposure compared to other groups. This unexpected result is accompanied by higher cell death rates, suggesting that short-term viral control may be linked to excessive inflammation, which can be potentially harmful in the long term. In contrast, cells from CPHIV-VS_≥5_ are predicted to maintain lower viral concentrations while limiting cell death. This could indicate a more effective and sustainable short-term immune response in CPHIV-VS_≥5_. CHU_≥5_ and CPHIV-VS_<5_ have the highest predicted levels of viral concentration and infected cells and high levels of cell death. Our model predicts distinct patterns of cell death across participant groups. CD8 T cell death contributes the most to cell death in CPHIV-VA while contributing the least in both age groups of CHU and CPHIV-VS which are dominated by CD4 T cell death. Cell death by excessive activation is the main death pathway in all groups, but HIV infection of CD4 T cells is much stronger in CHU and CPHIV_<5_ compared to other groups. Lastly, our results suggest that despite the impaired functionality of exhausted CD8 T cells (in proliferation, cytokine secretion and killing), large enough numbers of exhausted CD8 T cells can overcome these limitations. Our simulations therefore highlight unique patterns of immune responses to short-term HIV exposure resulting from unique and complex participant-specific immune profiles. We identify immune response patterns that vary with both age and HIV status.

Simulations for CPHIV-VS_<5_ participants predict higher viral concentration and cell death as compared to older participants who are virally suppressed. This suggests that CPHIV-VS_<5y_ are mounting an ineffective inflammatory response to the new HIV exposure. This is consistent with evolving immunologic maturity over the first 5 years of life (60–62). Neonatal monocytes have reduced production of pro-inflammatory cytokines compared to adult monocytes, including TNFα and IFN-γ (63–66). TNF-α remains low in infants up until age 5 years(67) then increases until it reaches peak concentrations at age 14 years that are higher than adult levels, then drops to adult levels(68). IFN-γ was seen to be in normal adult range from age 3–17 years(68) which leads to the general assumption that increased levels indicate inflammation. Overall, CPHIV-VS_<5y_ immune response patterns strongly resemble the age-matched healthy control group of CHU. Our simulations suggest that CPHIV who become virally suppressed before they reach 5 years in age, have limited immune exhaustion which enables them to respond to new infection challenges in a similar manner to children without HIV.

In contrast, cells from CPHIV-VS_≥5y_ are predicted to have lower viral load and lower cell death compared to their younger counterparts as well as CPHIV-VA. This suggests that CPHIV-VS_≥5y_ are able to mount an effective immune response that can limit viral replication with less host cell death. However, in our analysis this immune response is associated with more cell death from excessive activation rather than cytotoxic killing of infected cells. Thus, it appears that cells from CPHIV-VS_≥5y_ can indeed mount a more inflammatory response (more immune cell activation), but that this is not necessarily associated with improved killing of infected cells. This observation may be one mechanism that contributes to chronic inflammation reported in longer-term infections. Viral suppression in infants and young children under 5 years initiating ART takes longer than in older adults, which has been attributed to immunologic immaturity (69,70). In a Canadian cohort, children above age 5 years starting ART had a higher probability of viral rebound, which has also been seen in other cohorts of children (71,72). Indeed, the cell death patterns for CPHIV-VS_≥5y_ resemble those from CPHIV-VA more closely than CPHIV-VS_<5_. This suggests that despite viral suppression, the length of time since infection in CPHIV-VS_≥5y_ participants has been long enough that the cell death mechanisms have started to shift more closely to virally active patterns, even though the metrics of exhaustion still show much lower levels compared to virally active participants.

Finally, CPHIV-VA show very little differences between age groups, suggesting that the impacts of active viral replication are larger than the impacts of age. Our results indicate that in the short-term, viral replication is limited due to large amounts of cell death that mostly stems from excessive activation of macrophages, CD4 and CD8 T cells. Many forms of programmed cell death have been associated with HIV infection, including apoptotic and nonapoptotic pathways(73). The induction of apoptosis with pro-apoptotic TNF-derived peptides is associated with reduced HIV-associated cell death and reduced viral load(74). Meanwhile, nonapoptotic pathways, like necroptosis and pyroptosis, are thought to be host-detrimental leading to increased cell death and immunosuppression(73). Activation-induced cell death of T cells is found to be higher in persons with HIV, compared to healthy controls, with a reduction following ART (75–78). This evidence supports simulation results that CPHIV have more activation-associated deaths compared to CHU, and CPHIV-VS has fewer activation-induced deaths than CPHIV-VA. Surprisingly, our simulations predict that a sizable portion of exhausted CD8 T cells continue to contribute to CD8 T cell cytotoxic killing, proliferation, and cytokine secretion in these short-term infections. However, the large amounts of cell death from excessive activation indicate that these exhausted cells are likely to be depleted as the infection progresses to longer periods, possibly leading to increased viral replication long-term.

As with all computational models, there are limitations associated with necessary simplifying assumptions. We acknowledge that our short-term simulations of exposure to new HIV infections (emulating *in vitro* infection assays) are limited to making predictions about short-term *in vitro* dynamics, and therefore do not account for other dynamics that emerge *in vivo* over longer periods. Future work will incorporate methods presented here into simulations of longer-term *in vitro* experiments including periodic cell supplementations, and into our existing models of *in vivo* infections(79,80). This will inform both *in vitro* experimental design as well as understanding of *in vivo* infection dynamics. Additional limitations include necessary assumptions about how to map the IR markers to simulation mechanisms, including that IR expression is linearly correlated with function impairment as well as having any ability at all to perform a function and which functions are impacted by IR expression. We believe that these assumptions are reasonable given current knowledge (5,33), and can be adapted as more data on the impact of individual IR markers become available.

In short, our computational simulations integrating individualized immune profiles with known mechanisms and parameters allows us to predict the combined outcome of complex immune responses to new HIV exposure. Our simulations suggest that partially-functional exhausted CD8+ T cells can contribute to short-term viral suppression in CPHIV. Further, our results indicate that viral suppression in CPHIV can allow immune responses similar to CHU, but only in CPHIV_<5_. These findings could inform immunotherapeutic strategies, HIV cure studies, and HIV early treatment studies.

## Figures and Tables

**Table 1:** Demographic and clinical characteristics of participants with immune profiles used in simulations.

	Age 0–5 years				Age 5–20 years			
	CHU	CPHIV-VS	CPHIV-VA	p-value	CHU	CPHIV-VS	CPHIV-VA	p-value
n	12	13	24		20	19	28	
Age (years)^a^	2.6 (1.5 – 3.3)	3.2 (1.7 – 3.6)	2.4 (1.5 – 3.0)	NS^b^	13.2 (10.6 – 17.3)	11.84(8.3 – 13)	11.7 (9.1 – 16.5)	NS^b^
Female sex, No. (%)	10 (83.3%)	8 (61.5%)	9 (37.5%)	p<0.002^c^	4 (20%)	10 (52.6%)	16 (57.1%)	p=0.01^c^
HIV copies/mL^a^		110 (110–110)	65725 (3099 – 257400)	p<0.001^b^		110 (110–110)	52058 (6738 – 172700)	p<0.001^b^
Absolute CD4 count (cells/mm^3^)^a^	1595 (1314 – 2116)	1703 (1220 – 2194)	1552 (1065 – 2021)	NS^b^	779 (591 – 931)	957 (674–1216)	498 (313 – 790)	p<0.001^b^

(a) Median values with interquartile range

(b) Kruskal-Wallis test

(c) Chi-squared test

(NS) not significant; Significance determined at the threshold of p-value < 0.05

**Table 2:** Mapping of clinical markers to simulation mechanisms.

Clinical Marker	Simulation mechanism
CXCR3+	T cell has a greater individual probability of moving
CCR5+	CD4 T cell can be infected by HIV
HIV-specific	Antigen specific T cell that can perform actions based on other markers (activate macrophages, proliferate)
IFNγ+ TNFα+	T cell can secrete these cytokines
CD38+	T cell will be activated at start of simulation
Ki67+	T cell will be activated at start of simulation and has the ability to proliferate
Perforin+ Granzyme B+	CD8 T cell that has the ability to kill HIV-infected cells
PD1+ CD160+ TIM3+	Inhibitory markers (IRs)/exhaustion markers on T cell

**Table 3:** Original parameter ranges used to identify 20 representative parameter sets for a robust HIV infection. Parameter ranges were determined based on available literature or defined based on biological feasibility if no literature values could be found and then estimated using LHS.

Name	Value	Units	Reference
**CD4 Parameters affected by IRs**			
ActivatedCD4IFNSecretion*	5–40	Molecules/cells/s	(40,41)
ActivatedCD4TNFSecretion*	5–40	Molecules/cells/s	(40,41)
proliferationProbabilityCD4*	0.1–1	Per timestep	
**CD8 Parameters affected by IRs**			
ActivatedCD8IFNSecretion*	5–40	Molecules/cells/s	(40,41)
ActivatedCD8TNFSecretion*	5–40	Molecules/cells/s	(40,41)
CD8HIVSpecificKillProbability*	0.1–1	Per timestep	
proliferationProbabilityCD8*	0.1–1	Per timestep	
**HIV Parameters**			
startingFreeVirus*	1–100,000	Concentration/simulation volume	e
eclipsePhaseForVirusProduction	24	hours	(42)
eclipsePhaseForVirusProductionVariance	0.2		
hivCellCellInfectionProbability*	0.25–1	Per interaction	e
hivFreeVirusInfectionProbability*	0.25–1	Per interaction	e
hivProductionRateVariance	0.1		
maxHIVInfectedCellLifespan	2.2	days	(38)
maxHIVInfectedCellLifespanVariance	0.2		
viralProductionRatePerDayCD4	5	10^4^ virions/day	(43)
viralProductionRatePerDayMacro	5	10^4^ virions /day	(43)
virusDegradationRate	2.67	10^−5^ s^−1^	(38) (free virions in plasma lifespan of 0.3 days turned into rate)
virusDiffusionCoefficient	4.38	10^−8^ cm^2^/s	(44) (Stokes Einstein #)
**CD4 Parameters not affected by IRs**			
activatedMovementProbabilityCD4*+	0.1–1	Per timestep	e
baseMovementProbabilityCD4*+	0.1–1	Per timestep	e
CD4ActivationProbability*	0.1–1	Per interaction	e
CD4DeactivationProbability*	0–0.2	Per timestep	
cd4PopulationDoublingTime*	4–16	Hours	(45–47)
cd4PopulationActivatedLifespan	4	Days	(45–48)
cd4PopulationMaxLifespan	340	Days	(49)
cd4DoublingTimeVariance	0.1		
cd4LifeSpanVariance	0.1		
maximumCD4Generations*	4–10	Generations	(45–47,50,51)
hivSpecificNaiveCD4Fraction	0.02		
hivSpecificMemoryCD4Fraction	0.02		
percentCCR5NaiveCD4s*	0.021–0.83		(52)
**CD8 Parameters not affected by IRs**			
activatedMovementProbabilityCD8*+	0.1–1	Per timestep	e
baseMovementProbabilityCD8*+	0.1–1	Per timestep	e
CD8HIVSpecificActivationProbability*	0.2–1	Per interaction	e
CD8HIVSpecificDeactivationProbability*	0–0.2	Per timestep	
cd8PopulationDoublingTime*	4–16	Hours	(45–47)
cd8PopulationMaxActivatedLifespan	4	Days	(45–48)
cd8PopulationMaxLifespan	340	Days	(49)
cd8DoublingTimeVariance	0.1		
cd8LifeSpanVariance	0.1		
maximumCD8Generations*	4–20	Generations	(45–47,50,51)
hivSpecificCD8Fraction	0.02		
hivSpecificCD8NaiveFraction	0.02		
**Macrophage Parameters**			
ActivatedMacrophageTNFSecretion*	5–40	Molecules/cell/s	(40,41)
activatedMovementProbabilityMacro*	0.1–1	Per timestep	e
baseMovementProbabilityMacro*	0.1–1	Per timestep	e
macrophageLifeSpanVariance	0.1		
macrophagePopulationMaxActivatedLifespan	10	Days	(28)
macrophagePopulationMaxLifespan	100	Days	
nfkbSpan*	0.11–115	Hours	(29)
nfkbVariance	0.1		
stat1Span*	0.11–115	Hours	(29)
stat1Variance	0.1		
TNFthresholdForNFkBActivation*	10–500	Molecules	e
IFNthresholdForStat1Activation*	10–500	Molecules	e
percentCCR5Macs*	0.242–0.902		(52)
activatedMacrophageProportion*	0.05–0.5		
**Cytokine/General Immune Cell Parameters**			
InitialPBMCs*	6000–10000		e
IFNDegradationRatePerSecond*	0.96–10	s^−1^	(53–57)
IFNDiffusionCoefficient*	0.1–1	cm^2^/s	(53–55,57–59)
TNFDegradationRatePerSecond*	0.96–10	s^−1^	(53–57)
TNFDiffusionCoefficient*	0.1–1	cm^2^/s	(53–55,57–59)
TNFthresholdForImmuneCellMovement*	10–500	Molecules	e
Kapop*	9.93E-05 – 0.00993365		e
Kd*	0.5450045 – 54.5004503	10^6^	e
tauapop*	0.06 – 0.24		e

*: 20 parameter sets determined from the initial LHS range.

+: parameter is affected by CXCR3;

e: no literature value could be identified therefore this parameter was estimated to be biologically feasible and estimated through LHS.

**Table 4:** Statistical comparison between viral participant groups for each age participant group corresponding to Fig. 3a, b, and c respectively.

a)	Day 2		Day 3		Day 4	
	< 5yr	≥ 5yr	< 5yr	≥ 5yr	< 5yr	≥ 5yr
CHU vs CPHIV_VS_	**	***		***		***
CHU vs CPHIV_VA_		*	***	***	***	***
CPHIV_VA_ vs CPHIV_VS_	***		***	***	***	***
b)						
CHU vs CPHIV_VS_	*	***	*	***		***
CHU vs CPHIV_VA_				**	***	***
CPHIV_VA_ vs CPHIV_VS_		***	***		***	***
c)						
CHU vs CPHIV_VS_	***	***				
CHU vs CPHIV_VA_	***					
CPHIV_VA_ vs CPHIV_VS_		***				

Significance is determined with a two tailed t test between age-matched viral participant groups at α= 0.01.

*** p <= 1e-4,

** p<=1e-3,

* p<=0.01.

## Data Availability

Supplemental material, including participant-specific and non-participant specific parameters, MATLAB data and graphing scripts, are archived on Zenodo (10.5281/zenodo.17148547). The Repast model and accompanying parameter file to run the code can be found at: https://github.itap.purdue.edu/ElsjePienaarGroup/PediatricHIVExhaustionModel.

## References

[1] UNAIDS, UNAIDS Global AIDS update. The path that ends AIDS [Internet]. 2023. Available from: http://www.wipo.int/amc/en/mediation/rules

[2] Eckard AR, Rosebush JC, Lee ST, O’Riordan MA, Habib JG, Daniels JE, Increased Immune Activation and Exhaustion in HIV-infected Youth. Pediatric Infectious Disease Journal [Internet]. 2016 Dec;35(12):e370–7. Available from: https://journals.lww.com/00006454-201612000-00012 27626922 10.1097/INF.0000000000001326 PMC5157155

[3] Foldi J, Kozhaya L, McCarty B, Mwamzuka M, Marshed F, Ilmet T, HIV-Infected Children Have Elevated Levels of PD-1+ Memory CD4 T Cells With Low Proliferative Capacity and High Inflammatory Cytokine Effector Functions. J Infect Dis [Internet]. 2017 Sep 15;216(6):641–50. Available from: https://academic.oup.com/jid/article/216/6/641/4036242 28934428 10.1093/infdis/jix341 PMC5853680

[4] Ssewanyana I, Baker CAR, Ruel T, Bousheri S, Kamya M, Dorsey G, The Distribution and Immune Profile of T Cell Subsets in HIV-Infected Children from Uganda. AIDS Res Hum Retroviruses [Internet]. 2009 Jan;25(1):65–71. Available from: http://www.liebertpub.com/doi/10.1089/aid.2008.0138 19182922 10.1089/aid.2008.0138 PMC2858297

[5] Tailor J, Foldi J, Generoso M, McCarty B, Alankar A, Kilberg M, Disease Progression in Children With Perinatal Human Immunodeficiency Virus Correlates With Increased PD-1+ CD8 T Cells That Coexpress Multiple Immune Checkpoints. J Infect Dis [Internet]. 2021 Nov 22;224(10):1785–95. Available from: https://academic.oup.com/jid/article/224/10/1785/6231805 33864071 10.1093/infdis/jiab204 PMC9631235

[6] International Aids Society. Ias 2017 conference report [Internet]. Violari, A in. Paris, France; 2017. Available from: https://www.iasociety.org/sites/default/files/Conference reports2011/IAS2017_conference_report.pdf

[7] Greenberg RG, Corneli A, Bradley J, Farley J, Jafri HS, Lin L, Perceived barriers to pediatrician and family practitioner participation in pediatric clinical trials: Findings from the Clinical Trials Transformation Initiative. Contemp Clin Trials Commun [Internet]. 2018 Mar;9(September 2017):7–12. Available from: 10.1016/j.conctc.2017.11.006 29696219 PMC5898553

[8] Alvarez P, Mwamzuka M, Marshed F, Kravietz A, Ilmet T, Ahmed A, Immune activation despite preserved CD4 T cells in perinatally HIV-infected children and adolescents. Nixon DF, editor. PLoS One [Internet]. 2017 Dec 29;12(12):e0190332. Available from: https://dx.plos.org/10.1371/journal.pone.0190332 29287090 10.1371/journal.pone.0190332 PMC5747457

[9] El Hed A, Khaitan A, Kozhaya L, Manel N, Daskalakis D, Borkowsky W, Susceptibility of Human Th17 Cells to Human Immunodeficiency Virus and Their Perturbation during Infection. J Infect Dis [Internet]. 2010 Mar 15;201(6):843–54. Available from: https://academic.oup.com/jid/article-lookup/doi/10.1086/651021 20144043 10.1086/651021 PMC2849315

[10] Khaitan A, Kilberg M, Kravietz A, Ilmet T, Tastan C, Mwamzuka M, HIV-Infected Children Have Lower Frequencies of CD8+ Mucosal-Associated Invariant T (MAIT) Cells that Correlate with Innate, Th17 and Th22 Cell Subsets. Sandberg JK, editor. PLoS One [Internet]. 2016 Aug 25;11(8):e0161786. Available from: https://dx.plos.org/10.1371/journal.pone.0161786 27560150 10.1371/journal.pone.0161786 PMC4999196

[11] Khaitan A, Kravietz A, Mwamzuka M, Marshed F, Ilmet T, Said S, FOXP3+Helios+ Regulatory T Cells, Immune Activation, and Advancing Disease in HIV-Infected Children. JAIDS Journal of Acquired Immune Deficiency Syndromes [Internet]. 2016 Aug 15;72(5):474–84. Available from: https://journals.lww.com/00126334-201608150-00002 27003495 10.1097/QAI.0000000000001000 PMC4942350

[12] Generoso M, Álvarez P, Kravietz A, Mwamzuka M, Marshed F, Ahmed A, High soluble CD163 levels correlate with disease progression and inflammation in Kenyan children with perinatal HIVinfection. AIDS [Internet]. 2020 Jan 1;34(1):33–8. Available from: https://journals.lww.com/10.1097/QAD.0000000000002378 31567161 10.1097/QAD.0000000000002378

[13] McCarty B, Mwamzuka M, Marshed F, Generoso M, Alvarez P, Ilmet T, Low Peripheral T Follicular Helper Cells in Perinatally HIV-Infected Children Correlate With Advancing HIV Disease. Front Immunol [Internet]. 2018 Aug 24;9(August):1–12. Available from: https://www.frontiersin.org/article/10.3389/fimmu.2018.01901/full 30197641 10.3389/fimmu.2018.01901 PMC6117426

[14] Hoerter A, Petrucciani A, Bonifacio J, Arnett E, Schlesinger LS, Pienaar E. Timing matters in macrophage/CD4+ T cell interactions: an agent-based model comparing Mycobacterium tuberculosis host-pathogen interactions between latently infected and naïve individuals. Bartko A, editor. mSystems [Internet]. 2025 Feb 7;(February 2025). Available from: https://journals.asm.org/doi/10.1128/msystems.01290-24 10.1128/msystems.01290-24 PMC11915833 39918314

[15] Petrucciani A, Hoerter A, Kotze L, Du Plessis N, Pienaar E. Agent-based model predicts that layered structure and 3D movement work synergistically to reduce bacterial load in 3D in vitro models of tuberculosis granuloma. Vega N, editor. PLoS Comput Biol [Internet]. 2024 Jul 12;20(7):e1012266. Available from: 10.1371/journal.pcbi.1012266 38995971 PMC11288457

[16] Petrucciani A, Hoerter A, Kotze L, Du Plessis N, Pienaar E. In silico agent-based modeling approach to characterize multiple in vitro tuberculosis infection models. Puniya BL, editor. PLoS One [Internet]. 2024 Mar 22;19(3):e0299107. Available from: http://biorxiv.org/content/early/2023/03/14/2023.03.13.532338.abstract 38517920 10.1371/journal.pone.0299107 PMC10959380

[17] Barros-Becker F, Lam PY, Fisher R, Huttenlocher A. Live imaging reveals distinct modes of neutrophil and macrophage migration within interstitial tissues. J Cell Sci [Internet]. 2017 Jan 1 [cited 2022 Nov 2];130(22):3801–8. Available from: 10.1242/jcs.206128 28972134 PMC5702045

[18] Pixley FJ. Macrophage Migration and Its Regulation by CSF-1. Int J Cell Biol [Internet]. 2012 [cited 2022 Nov 2];2012:1–12. Available from: http://www.hindawi.com/journals/ijcb/2012/501962/10.1155/2012/501962 PMC3296313 22505929

[19] Grabher C, Cliffe A, Miura K, Hayflick J, Pepperkok R, Rørth P, Birth and life of tissue macrophages and their migration in embryogenesis and inflammation in medaka. J Leukoc Biol [Internet]. 2007 Jan 1 [cited 2022 Nov 2];81(1):263–71. Available from: https://onlinelibrary.wiley.com/doi/full/10.1189/jlb.0806526 17046968 10.1189/jlb.0806526

[20] Bzymek R, Horsthemke M, Isfort K, Mohr S, Tjaden K, Müller-Tidow C, Real-time two- and three-dimensional imaging of monocyte motility and navigation on planar surfaces and in collagen matrices: roles of Rho. Sci Rep [Internet]. 2016 Apr 28 [cited 2022 Nov 2];6(1):25016. Available from: https://www.nature.com/articles/srep25016 27122054 10.1038/srep25016 PMC4848558

[21] North MJ, Collier NT, Ozik J, Tatara ER, Macal CM, Bragen M, Complex adaptive systems modeling with Repast Simphony. Complex Adaptive Systems Modeling [Internet]. 2013 Dec 13;1(1):3. Available from: http://www.casmodeling.com/content/2/1/6http://www.casmodeling.com/content/2/1/6

[22] Weathered C, Pennington K, Escalante P, Pienaar E. The Role of Biofilms, Bacterial Phenotypes, and Innate Immune Response in Mycobacterium avium Colonization to Infection. J Theor Biol [Internet]. 2022;534:110949. Available from: 10.1016/j.jtbi.2021.110949 34717938

[23] Cilfone NA, Kirschner DE, Linderman JJ. Strategies for Efficient Numerical Implementation of Hybrid Multi-scale Agent-Based Models to Describe Biological Systems. Cell Mol Bioeng [Internet]. 2015 Mar 8;8(1):119–36. Available from: http://link.springer.com/10.1007/s12195-014-0363-6 26366228 10.1007/s12195-014-0363-6 PMC4564133

[24] Fallahi-Sichani M, El-Kebir M, Marino S, Kirschner DE, Linderman JJ. Multiscale Computational Modeling Reveals a Critical Role for TNF-α Receptor 1 Dynamics in Tuberculosis Granuloma Formation. The Journal of Immunology. 2011;186(6):3472–83. 21321109 10.4049/jimmunol.1003299 PMC3127549

[25] Rock KL, Reits E, Neefjes J. Present Yourself! By MHC Class I and MHC Class II Molecules. Trends Immunol [Internet]. 2016 Nov;37(11):724–37. Available from: 10.1016/j.it.2016.08.010 27614798 PMC5159193

[26] Liu T, Zhang L, Joo D, Sun SC. NF-κB signaling in inflammation. Signal Transduct Target Ther [Internet]. 2017 Jul 14;2(1):17023. Available from: https://www.nature.com/articles/sigtrans201723 29158945 10.1038/sigtrans.2017.23 PMC5661633

[27] Dorrington MG, Fraser IDC. NF-κB Signaling in Macrophages: Dynamics, Crosstalk, and Signal Integration. Front Immunol [Internet]. 2019 Apr 9;10(APR). Available from: https://www.frontiersin.org/article/10.3389/fimmu.2019.00705/full 10.3389/fimmu.2019.00705 PMC6465568 31024544

[28] Fallahi-Sichani M, Kirschner DE, Linderman JJ. NF-κB Signaling Dynamics Play a Key Role in Infection Control in Tuberculosis. Front Physiol [Internet]. 2012;3(June):1–25. Available from: http://journal.frontiersin.org/article/10.3389/fphys.2012.00170/abstract 22685435 10.3389/fphys.2012.00170 PMC3368390

[29] Marino S, Cilfone NA, Mattila JT, Linderman JJ, Flynn JL, Kirschner DE. Macrophage polarization drives granuloma outcome during Mycobacterium tuberculosis infection. Infect Immun [Internet]. 2015 Jan 1 [cited 2021 May 24];83(1):324–38. Available from: http://dx.doi.org/10.1128 25368116 10.1128/IAI.02494-14 PMC4288886

[30] Groom JR, Luster AD. CXCR3 in T cell function. Exp Cell Res [Internet]. 2011 Mar;317(5):620–31. Available from: 10.1016/j.yexcr.2010.12.017 21376175 PMC3065205

[31] Kohlmeier JE, Cookenham T, Miller SC, Roberts AD, Christensen JP, Thomsen AR, CXCR3 Directs Antigen-Specific Effector CD4+ T Cell Migration to the Lung During Parainfluenza Virus Infection. The Journal of Immunology [Internet]. 2009 Oct 1;183(7):4378–84. Available from: https://journals.aai.org/jimmunol/article/183/7/4378/81758/CXCR3-Directs-Antigen-Specific-Effector-CD4-T-Cell 19734208 10.4049/jimmunol.0902022 PMC2757292

[32] Bevan MJ. Helping the CD8+ T-cell response. Nat Rev Immunol [Internet]. 2004 Aug;4(8):595–602. Available from: https://www.nature.com/articles/nri1413 15286726 10.1038/nri1413

[33] Wherry EJ, Kurachi M. Molecular and cellular insights into T cell exhaustion. Nat Rev Immunol [Internet]. 2015 Aug 24;15(8):486–99. Available from: https://www.nature.com/articles/nri3862 26205583 10.1038/nri3862 PMC4889009

[34] Wagle M V., Vervoort SJ, Kelly MJ, Van Der Byl W, Peters TJ, Martin BP, Antigen-driven EGR2 expression is required for exhausted CD8+ T cell stability and maintenance. Nat Commun [Internet]. 2021 May 13;12(1):2782. Available from: https://www.nature.com/articles/s41467-021-23044-9 33986293 10.1038/s41467-021-23044-9 PMC8119420

[35] Stevenson M, Stanwick TL, Dempsey MP, Lamonica CA. HIV-1 replication is controlled at the level of T cell activation and proviral integration. EMBO J [Internet]. 1990 May;9(5):1551–60. Available from: https://onlinelibrary.wiley.com/doi/10.1002/j.1460-2075.1990.tb08274.x 2184033 10.1002/j.1460-2075.1990.tb08274.x PMC551849

[36] Zack JA, Arrigo SJ, Weitsman SR, Go AS, Haislip A, Chen ISY. HIV-1 entry into quiescent primary lymphocytes: Molecular analysis reveals a labile, latent viral structure. Cell [Internet]. 1990 Apr;61(2):213–22. Available from: https://linkinghub.elsevier.com/retrieve/pii/009286749090802L 2331748 10.1016/0092-8674(90)90802-l

[37] Virgilio MC, Collins KL. The Impact of Cellular Proliferation on the HIV-1 Reservoir. Viruses [Internet]. 2020 Jan 21;12(2):127. Available from: https://www.mdpi.com/1999-4915/12/2/127 31973022 10.3390/v12020127 PMC7077244

[38] Perelson AS, Neumann AU, Markowitz M, Leonard JM, Ho DD. HIV-1 Dynamics in Vivo: Virion Clearance Rate, Infected Cell Life-Span, and Viral Generation Time. Science (1979) [Internet]. 1996 Mar 15;271(5255):1582–6. Available from: https://www.sciencemag.org/lookup/doi/10.1126/science.271.5255.1582 10.1126/science.271.5255.1582 8599114

[39] Marino S, Hogue IB, Ray CJ, Kirschner DE. A methodology for performing global uncertainty and sensitivity analysis in systems biology. J Theor Biol. 2008;254(1):178–96. 18572196 10.1016/j.jtbi.2008.04.011 PMC2570191

[40] Han Q, Bradshaw EM, Nilsson B, Hafler DA, Love JC. Multidimensional analysis of the frequencies and rates of cytokine secretion from single cells by quantitative microengraving. Lab Chip [Internet]. 2010;10(11):1391. Available from: http://xlink.rsc.org/?DOI=b926849a 20376398 10.1039/b926849a PMC3128808

[41] Lu Y, Chen JJ, Mu L, Xue Q, Wu Y, Wu PH, High-Throughput Secretomic Analysis of Single Cells to Assess Functional Cellular Heterogeneity. Anal Chem [Internet]. 2013 Feb 19;85(4):2548–56. Available from: https://www.worldscientific.com/worldscibooks/10.1142/9614 23339603 10.1021/ac400082e PMC3589817

[42] Kakizoe Y, Nakaoka S, Beauchemin CAA, Morita S, Mori H, Igarashi T, A method to determine the duration of the eclipse phase for in vitro infection with a highly pathogenic SHIV strain. Sci Rep [Internet]. 2015;5(January):1–14. Available from: 10.1038/srep10371 PMC4440524 25996439

[43] De Boer RJ, Ribeiro RM, Perelson AS. Current Estimates for HIV-1 Production Imply Rapid Viral Clearance in Lymphoid Tissues. Fraser C, editor. PLoS Comput Biol [Internet]. 2010 Sep 2;6(9):e1000906. Available from: https://dx.plos.org/10.1371/journal.pcbi.1000906 20824126 10.1371/journal.pcbi.1000906 PMC2932679

[44] Imle A, Kumberger P, Schnellbächer ND, Fehr J, Carrillo-Bustamante P, Ales J, Experimental and computational analyses reveal that environmental restrictions shape HIV-1 spread in 3D cultures. Nat Commun [Internet]. 2019;10(1):2144. Available from: http://www.nature.com/articles/s41467-019-09879-3 31086185 10.1038/s41467-019-09879-3 PMC6514199

[45] Ziraldo C, Gong C, Kirschner DE, Linderman JJ. Strategic priming with multiple antigens can yield memory cell phenotypes optimized for infection with mycobacterium tuberculosis: A computational study. Front Microbiol. 2016;6(JAN):1–21. 10.3389/fmicb.2015.01477 PMC4701940 26779136

[46] Linderman JJ, Riggs T, Pande M, Miller M, Marino S, Kirschner DE. Characterizing the Dynamics of CD4+ T Cell Priming within a Lymph Node. The Journal of Immunology. 2010 Mar 15;184(6):2873–85. 20154206 10.4049/jimmunol.0903117 PMC3153313

[47] Gong C, Linderman JJ, Kirschner D. Harnessing the heterogeneity of T cell differentiation fate to fine-tune generation of effector and memory T cells. Front Immunol. 2014;5(FEB):1–15. 24600448 10.3389/fimmu.2014.00057 PMC3928592

[48] Gossel G, Hogan T, Cownden D, Seddon B, Yates AJ. Memory CD4 T cell subsets are kinetically heterogeneous and replenished from naive T cells at high levels. Elife. 2017 Mar 10;6:1–30. 10.7554/eLife.23013 PMC5426903 28282024

[49] Borghans JAM, Tesselaar K, de Boer RJ. Current best estimates for the average lifespans of mouse and human leukocytes: reviewing two decades of deuterium-labeling experiments. Immunol Rev [Internet]. 2018 Sep 11;285(1):233–48. Available from: https://onlinelibrary.wiley.com/doi/10.1111/imr.12693 30129193 10.1111/imr.12693

[50] Foulds KE, Zenewicz LA, Shedlock DJ, Jiang J, Troy AE, Shen H. Cutting Edge: CD4 and CD8 T Cells Are Intrinsically Different in Their Proliferative Responses. The Journal of Immunology. 2002;168(4):1528–32. 11823476 10.4049/jimmunol.168.4.1528

[51] Obst R. The timing of T cell priming and cycling. Front Immunol. 2015;6(NOV):1–10. 26594213 10.3389/fimmu.2015.00563 PMC4633513

[52] Lee B, Sharron M, Montaner LJ, Weissman D, Doms RW. Quantification of CD4, CCR5, and CXCR4 levels on lymphocyte subsets, dendritic cells, and differentially conditioned monocyte-derived macrophages. Proc Natl Acad Sci U S A. 1999;96(9):5215–20. 10220446 10.1073/pnas.96.9.5215 PMC21844

[53] Fallahi-Sichani M, El-Kebir M, Marino S, Kirschner DE, Linderman JJ. Multiscale Computational Modeling Reveals a Critical Role for TNF-α Receptor 1 Dynamics in Tuberculosis Granuloma Formation. The Journal of Immunology. 2011;186(6):3472–83. 21321109 10.4049/jimmunol.1003299 PMC3127549

[54] Cilfone NA, Perry CR, Kirschner DE, Linderman JJ. Multi-Scale Modeling Predicts a Balance of Tumor Necrosis Factor-α and Interleukin-10 Controls the Granuloma Environment during Mycobacterium tuberculosis Infection. PLoS One. 2013;8(7). 10.1371/journal.pone.0068680 PMC3711807 23869227

[55] Pollmächer J, Figge MT. Deciphering chemokine properties by a hybrid agent-based model of Aspergillus fumigatus infection in human alveoli. Front Microbiol. 2015;6(MAY):1–14. 26074897 10.3389/fmicb.2015.00503 PMC4446573

[56] Bowness R, Chaplain MAJ, Powathil GG, Gillespie SH. Modelling the effects of bacterial cell state and spatial location on tuberculosis treatment: Insights from a hybrid multiscale cellular automaton model. J Theor Biol [Internet]. 2018;446:87–100. Available from: 10.1016/j.jtbi.2018.03.006 29524441 PMC5901892

[57] Ray JCJ, Flynn JL, Kirschner DE. Synergy between Individual TNF-Dependent Functions Determines Granuloma Performance for Controlling Mycobacterium tuberculosis Infection. The Journal of Immunology [Internet]. 2009 Mar 15;182(6):3706–17. Available from: https://journals.aai.org/jimmunol/article/182/6/3706/103675/Synergy-between-Individual-TNF-Dependent-Functions 19265149 10.4049/jimmunol.0802297 PMC3182770

[58] Ross AE, Pompano RR. Diffusion of cytokines in live lymph node tissue using microfluidic integrated optical imaging. Anal Chim Acta. 2018;1000:205–13. 29289312 10.1016/j.aca.2017.11.048

[59] Francis K, Palsson BO. Effective intercellular communication distances are determined by the relative time constants for cyto/chemokine secretion and diffusion. Proceedings of the National Academy of Sciences. 1997 Nov 11;94(23):12258–62. 10.1073/pnas.94.23.12258 PMC24899 9356436

[60] Tobin NH, Aldrovandi GM. Immunology Of Pediatric HIV Infection. Immunol Rev. 2013;254(1):143–69. 23772619 10.1111/imr.12074 PMC3737605

[61] Dunn DT, Gibb DM, Duong T, Babiker AG, Bulterys M, Gabiano C, Short-term risk of disease progression in HIV-1-infected children receiving no antiretroviral therapy or zidovudine monotherapy: a meta-analysis. The Lancet [Internet]. 2003 Nov;362(9396):1605–11. Available from: https://linkinghub.elsevier.com/retrieve/pii/S0140673603147939 10.1016/s0140-6736(03)14793-9 14630440

[62] Newell ML, Giaquinto C, Rampon O, Giacomet V, De Rossi A, Grosch-Wörner I, Level and pattern of HIV-1-RNA viral load over age: differences between girls and boys? AIDS [Internet]. 2002 Jan;16(1):97–104. Available from: http://journals.lww.com/00002030-200201040-00012 11741167 10.1097/00002030-200201040-00012

[63] Levy O, Coughlin M, Cronstein BN, Roy RM, Desai A, Wessels MR. The Adenosine System Selectively Inhibits TLR-Mediated TNF-α Production in the Human Newborn. The Journal of Immunology [Internet]. 2006 Aug 1;177(3):1956–66. Available from: https://journals.aai.org/jimmunol/article/177/3/1956/220/The-Adenosine-System-Selectively-Inhibits-TLR 16849509 10.4049/jimmunol.177.3.1956 PMC2881468

[64] Levy O. Innate immunity of the newborn: Basic mechanisms and clinical correlates. Nat Rev Immunol. 2007;7(5):379–90. 17457344 10.1038/nri2075

[65] Levy O, Zarember KA, Roy RM, Cywes C, Godowski PJ, Wessels MR. Selective Impairment of TLR-Mediated Innate Immunity in Human Newborns: Neonatal Blood Plasma Reduces Monocyte TNF. J Immunol. 2004;173(2004):4627–34. 15383597 10.4049/jimmunol.173.7.4627

[66] Angelone DF, Wessels MR, Coughlin M, Suter EE, Valentini P, Kalish LA, Innate immunity of the human newborn is polarized toward a high ratio of IL-6/TNF-α production in vitro and in vivo. Pediatr Res. 2006;60(2):205–9. 16864705 10.1203/01.pdr.0000228319.10481.ea

[67] Berdat PA, Wehrle TJ, Küng A, Achermann F, Sutter M, Carrel TP, Age-specific analysis of normal cytokine levels in healthy infants. Clin Chem Lab Med. 2003;41(10):1335–9. 14580162 10.1515/CCLM.2003.204

[68] Sack U, Burkhardt U, Borte M, Schädlich H, Berg K, Emmrich F. Age-dependent levels of select immunological mediators in sera of healthy children. Clin Diagn Lab Immunol. 1998;5(1):28–32. 9455875 10.1128/cdli.5.1.28-32.1998 PMC121386

[69] Kakkar F, Lee T, Hawkes MT, Brophy J, Lindy S, Singer J, Challenges to achieving and maintaining viral suppression among children living with HIV. Aids. 2020;34(5):687–97. 31794519 10.1097/QAD.0000000000002454

[70] Shiau S, Abrams EJ, Arpadi SM, Kuhn L. Early antiretroviral therapy in HIV-infected infants: can it lead to HIV remission? Lancet HIV [Internet]. 2018 May;5(5):e250–8. Available from: https://linkinghub.elsevier.com/retrieve/pii/S2352301818300122 29739699 10.1016/S2352-3018(18)30012-2 PMC7487171

[71] Duong T, Judd A, Collins IJ, Doerholt K, Lyall H, Foster C, Long-term virological outcome in children on antiretroviral therapy in the UK and Ireland. Aids. 2014;28(16):2395–405. 25389551 10.1097/QAD.0000000000000438 PMC4210689

[72] Mu W, Bartlett AW, Bunupuradah T, Chokephaibulkit K, Kumarasamy N, Ly PS, Early and Late Virologic Failure after Virologic Suppression in HIV-Infected Asian Children and Adolescents. J Acquir Immune Defic Syndr (1988). 2019;80(3):308–15. 10.1097/QAI.0000000000001921 PMC6952284 30531299

[73] Paim AC, Badley AD, Cummins NW. Mechanisms of Human Immunodeficiency Virus-Associated Lymphocyte Regulated Cell Death. AIDS Res Hum Retroviruses. 2020;36(2):101–15. 31659912 10.1089/aid.2019.0213 PMC7044792

[74] Chen Q, Zhao Y, Zhang Y, Zhang J, Lu W, Chang CH, HIV associated cell death: Peptide-induced apoptosis restricts viral transmission. Front Immunol. 2023;14(February):1–9. 10.3389/fimmu.2023.1096759 PMC9992636 36911666

[75] Gougeon ML. To kill or be killed: How HIV exhausts the immune system. Cell Death Differ. 2005;12:845–54. 15832178 10.1038/sj.cdd.4401616

[76] Meyaard L, Miedema F. Programmed death of T cells in the course of HIV infection. Adv Exp Med Biol. 1995;374(July 1992):115–20. 7572385 10.1007/978-1-4615-1995-9_10

[77] Groux H, Torpier G, Monté D, Mouton Y, Capron A, Ameisen JC. Activation-induced death by apoptosis in CD4+ T cells from human immunodeficiency virus-infected asymptomatic individuals. Journal of Experimental Medicine. 1992;175(2):331–40. 1346269 10.1084/jem.175.2.331 PMC2119133

[78] Sloand EM, Kumar PN, Kim S, Chaudhuri A, Weichold FF, Young NS. Human immunodeficiency virus type 1 protease inhibitor modulates activation of peripheral blood CD4+ T cells and decreases their susceptibility to apoptosis in vitro and in vivo. Blood [Internet]. 1999;94(3):1021–7. Available from: 10.1182/blood.V94.3.1021.415k29_1021_1027 10419894

[79] Cody JW, Ellis-Connell AL, O’Connor SL, Pienaar E. Mathematical modeling indicates that regulatory inhibition of CD8+ T cell cytotoxicity can limit efficacy of IL-15 immunotherapy in cases of high pre-treatment SIV viral load. Ribeiro RM, editor. PLoS Comput Biol [Internet]. 2023 Aug 24;19(8):e1011425. Available from: 10.1371/journal.pcbi.1011425 37616311 PMC10482305

[80] Cody JW, Ellis-Connell AL, O’Connor SL, Pienaar E. Mathematical modeling of N-803 treatment in SIV-infected non-human primates. Ribeiro RM, editor. PLoS Comput Biol [Internet]. 2021 Jul 28;17(7):e1009204. Available from: 10.1371/journal.pcbi.1009204 34319980 PMC8351941

[81] Sfiligoi I, Bradley DC, Holzman B, Mhashilkar P, Padhi S, Wurthwein F. The Pilot Way to Grid Resources Using glideinWMS. In: 2009 WRI World Congress on Computer Science and Information Engineering [Internet]. IEEE; 2009. p. 428–32. (2; vol. 2). Available from: http://ieeexplore.ieee.org/document/5171374/

[82] OSG. OSPool [Internet]. OSG; 2006. Available from: https://osg-htc.org/services/open_science_pool.html

[83] OSG. Open Science Data Federation [Internet]. OSG; 2015. Available from: https://osdf.osg-htc.org/

[84] Pordes R, Petravick D, Kramer B, Olson D, Livny M, Roy A, The open science grid. J Phys Conf Ser [Internet]. 2007 Jul 1;78:012057. Available from: https://iopscience.iop.org/article/10.1088/1742-6596/78/1/012057

